# Use of the PROMIS Mobility score in assessing function in adolescents and adults previously affected by childhood hip disease

**DOI:** 10.1302/2633-1462.212.BJO-2021-0154.R1

**Published:** 2021-12-21

**Authors:** Weisang Luo, Mohammed S. Ali, Richard Limb, Christine Cornforth, Daniel C. Perry

**Affiliations:** 1 Trauma and Orthopaedics, Alder Hey Children's Hospital, Liverpool, UK; 2 Department of Women’s and Children’s Health, University of Liverpool Institute of Translational Medicine, Liverpool, UK; 3 Trauma and Orthopaedics, Aintree University Hospitals NHS Foundation Trust, Liverpool, UK; 4 Trauma and Orthopaedics, University of Liverpool and Alder Hey Children’s Hospital, Liverpool, UK

**Keywords:** PROMIS, Perthes' disease, Slipped Capital Femoral Epiphysis, Functional Outcome, Core Outcome set, hip diseases, Patient-Reported Outcomes Measurement Information System, arthritic hip, patient-reported outcome measures (PROMs), Legg-calve-perthes disease, Numeric Pain Rating Scale, hip, T-scores, EQ-5D-5L

## Abstract

**Aims:**

The Patient-Reported Outcomes Measurement Information System (PROMIS) has demonstrated faster administration, lower burden of data capture and reduced floor and ceiling effects compared to traditional Patient Reported Outcomes Measurements (PROMs). We investigated the suitability of PROMIS Mobility score in assessing physical function in the sequelae of childhood hip disease.

**Methods:**

In all, 266 adolscents (aged ≥ 12 years) and adults were identified with a prior diagnosis of childhood hip disease (either Perthes’ disease (n = 232 (87.2%)) or Slipped Capital Femoral Epiphysis (n = 34 (12.8%)) with a mean age of 27.73 years (SD 12.24). Participants completed the PROMIS Mobility Computer Adaptive Test, the Non-Arthritic Hip Score (NAHS), EuroQol five-dimension five-level questionnaire, and the Numeric Pain Rating Scale. We investigated the correlation between the PROMIS Mobility and other tools to assess use in this population and any clustering of outcome scores.

**Results:**

There was a strong correlation between the PROMIS Mobility and other established PROMs; NAHS (*r_s_* = 0.79; p < 0.001). There was notable clustering in PROMIS at the upper end of the distribution score (42.5%), with less seen in the NAHS (20.3%). However, the clustering was broadly similar between PROMIS Mobility and the comparable domains of the NAHS; function (53.6%), and activity (35.0%).

**Conclusion:**

PROMIS Mobility strongly correlated with other tools demonstrating convergent construct validity. There was clustering of physical function scores at the upper end of the distributions, which may reflect truncation of the data caused by participants having excellent outcomes. There were elements of disease not captured within PROMIS Mobility alone, and difficulties in differentiating those with the highest levels of function.

Cite this article: *Bone Jt Open* 2021;2(12):1089–1095.

## Introduction

The traditional means of classifying ‘outcomes’ of childhood hip diseases is almost entirely dependent upon the radiological appearance, along with the eventual need for a total hip arthroplasty.^1^ More recently, there is a focus on the outcomes of the disease considered more relevant to the patient by using functional scores to measure function and quality of life.^2,3^ Current literature recommends the use of Core Outcome Sets (COSs) to define how disease outcomes are measured, with multistakeholder groups defining the outcomes to be measured in any given disease area.^4-8^

To measure the different outcome domains within a COS, there is a plethora of patient-reported outcome measures (PROMs).^9^ Traditionally, PROMs are frequently time-consuming to complete, have high variability between instruments, have significant floor and ceiling effects, and produce a narrow scope of information.^10^ The limitations of legacy instruments has led to the development of Patient-Reported Outcomes Measurement Information System (PROMIS) by the US National Institute of Health to standardize outcome measures across a variety of health conditions with corresponding measurement tools.^11,12^ Of particular interest within orthopaedics are the PROMIS Physical Health Function (PF) tools, of which mobility (an individual’s self-perceived mobility characteristics), and upper limb (UE; an individual’s self-perceived upper-limb characteristics) tools are sub-domains. PROMIS tools can be used as a Computer Adaptive Test (CAT) or via Short Form, with CATs offering a tailored measurement question set based on responses, whereas Short Forms require all questions to be answered. PROMIS-CAT offer the broadest range of accurate scores.^13^ A systematic review concluded that there was a strong correlation between established PROM tools and PROMIS in 18 orthopaedic studies, with PROMIS demonstrating faster administration and greater applicability to a broad patient population while remaining highly reliable.^14^

Among children with Perthes' disease, PROMIS tools, including the child version of PROMIS Mobility, have demonstrated construct validity in this population,^15^ with accelerometer data among children with hip disease demonstrating convergent construct validity through the use of experimental data.^16^

We sought to test the convergent construct of the adult version of PROMIS-CAT Mobility in the functional assessment of mature individuals with a history of childhood hip disease. We investigated the convergent construct validity of PROMIS Mobility against the established Non-Arthritic Hip Score (NAHS), particularly considering the sub-domains. We also investigated the relationship with other health status measurement tools: the EuroQol five-dimension five-level (EQ-5D-5L) and the Numeric Pain Rating Scale (NRS). We assessed for the presence of ceiling and floor effects in all scores for this population to identify whether these instruments can distinguish between the range of patient outcomes observed. We hypothesised that there is a correlation between PROMIS and legacy instrument.

## Methods

Eligible participants with previous childhood hip disease were identified from the Alder Hey Perthes’ disease and Slipped Capital Femoral Epiphysis Registers. This was part of the Outcomes Research in Children’s Hip Disease (ORCHiD) study (IRAS ID 14201). Participants were aged 12 years or older. Inclusion in the registry was based on the criteria shown in Table I.

**Table I.** Disease specific inclusion criteria.

Perthes' disease	Slipped capital femoral epiphysis
Diagnosis made while skeletally immature. Any of the following radiological features within the femoral epiphysis: Flattening Sclerosis Fragmentation Collapse Re-ossification Features may be evident on plain radiographs, or MRI Resident within England, Scotland, or Wales Able to understand the study documentation No record of the following PRIOR to the first diagnosis Treatment for developmental hip dysplasia (not including double nappies) Chemotherapy for malignancy. Diagnosed sickle cell anaemia. Multiple epiphyseal dysplasia or spondyloepiphyseal dysplasia. A known coagulopathy Gaucher’s disease Same-sided hip fracture Hypothyroidism	Diagnosis made while skeletally immature. Radiological confirmation of displacement of the epiphysis relative to then metaphysis occurring at the proximal femoral physis. Treatment (surgery or activity restriction) following the diagnosis to stabilize the epiphysis. Able to understand the study documentation

Following the identification of eligible participants, an invitation pack was sent to potential participants. This included a letter inviting them to take part in the study, an information sheet, a paper consent form, and a prepaid envelope and link to an e-consent form. Participants and/or their parents were encouraged to contact the research team via email, phone, or post to discuss their participation and ask any questions.

Once the signed consent form from the participants and/or their parents have been received by the research team, the questionnaires were provided to the participants either electronically (automatically once consent was obtained), or by telephone, depending upon each individual participant preference. Any discrepancies were corrected by contacting the participant/participant’s parent over the telephone.

Data collection was between November 2017 to June 2019. Of the 856 patients invited to participate, 300 returned questionnaires. Complete responses were recorded for 291 participants (97%). Of those, 25 (8.6%) have had a history of hip arthroplasty, and therefore were excluded from analysis. Subsequently, 266 (91.4%) were included in the final statistical analysis.

### PROM selection

PROMIS Mobility v2.0 CAT was used, as a subset of the PROMIS Physical Function tools. All raw scores generated from PROMIS measures are translated into standardized T-scores. A T-score of 50 (standard deviation (SD) 10) represents the “general population mean”, referring to the mean score from the calibration sample that PROMIS was developed from.^17^

NAHS was selected due to its broad use in evaluating the young adult hip. NAHS is a widely used functional tool for assessing hip outcomes in adults without an obvious radiological diagnosis.^18^ It was designed for use in younger patients with higher physical demands and expectations than the degenerative joint disease population. This scoring system includes 20 multiple choice questions, each having the same five responses. There are four construct domains, with the final score calculated from the cumulative total. There are five questions for pain, four for mechanical symptoms, five for physical function, and six for activity-level.^18^ A score of 0 represents a hip without meaningful function, and 100 represents a ‘perfectly’ functioning hip.

EQ-5D-5L was selected in order to compare the participant’s general health status. EQ-5D-5L is a validated and standardized health-related quality of life measure consisting of five domains related to daily activities with a five-level answer choice.^19^ Respondents may answer from no problems (score = 1.0) to extreme impairment on all five dimensions (score = -0.594). A score < 0 indicates that quality of life is so poor, it is worse than death.

Numeric Rating Scale (NRS) for pain is a unidimensional measure of pain intensity in adults including those with chronic pain.^20^ Psychological and psychosocial factors from pain influence participant outcome, and was shown to be highly correlated to the visual analogue scale in patients with rheumatic and other chronic pain conditions.^21^ A score of 0 represents “no pain” while the other extreme (“worst imaginable pain”) is represented by a score of 10.

### Funding and ethics

Funding was obtained from Versus Arthritis (Funding reference 21356). Ethical approval was obtained from an UK regional Central Research Ethics Committee and Health Research Authority (HRA) (REC reference 17/ES/0113). This study has been reviewed and approved by independent members of the Perthes' Association and the Steps Charity.

### Statistical analysis

The PROMIS Mobility T-scores were compared with the index scores from the other PROMs by Spearman rank correlation coefficient. *r_s_* values of > 0.7 considered as a strong correlation, 0.5 to 0.7 a moderately strong correlation, 0.3 to 0.5 a weak correlation, and 0 to 0.3 corresponding to a negligible correlation.^22^ Each subset score for the NAHS was similarly correlated with PROMIS Mobility T-score. Floor and ceiling effects for each PROM were compared. The presence of clustering at the extremes was determined if the proportion of patients achieving the highest or lowest scores was > 15% of the best or worst achievable scores.^23^

Statistical analysis was performed using IBM SPSS statistical software (v. 26.0; IBM, USA), with p-values < 0.05 considered statistically significant.

## Results

In all, 266 participants completed all questionnaires (PROMIS Mobility, NAHS, EQ-5D-5L, and NRS). Participant demographics are summarized in Table II.

**Table II.** Patient demographics.

Variable	Data
Male, n (%)	202 (75.9)
Perthe's disease, n (%)	232 (87.2)
Slipped capital femoral epiphyses, n (%)	34 (12.8)
Mean age, yrs (SD; range)	27.73 (12.24; 12 to 57)

SD, standard deviation.

To calculate a score the PROMIS Mobility CAT, we asked an median of 8.5 questions (interquartile range (IQR) 4 to 12) per participant.

**Fig. 1:**
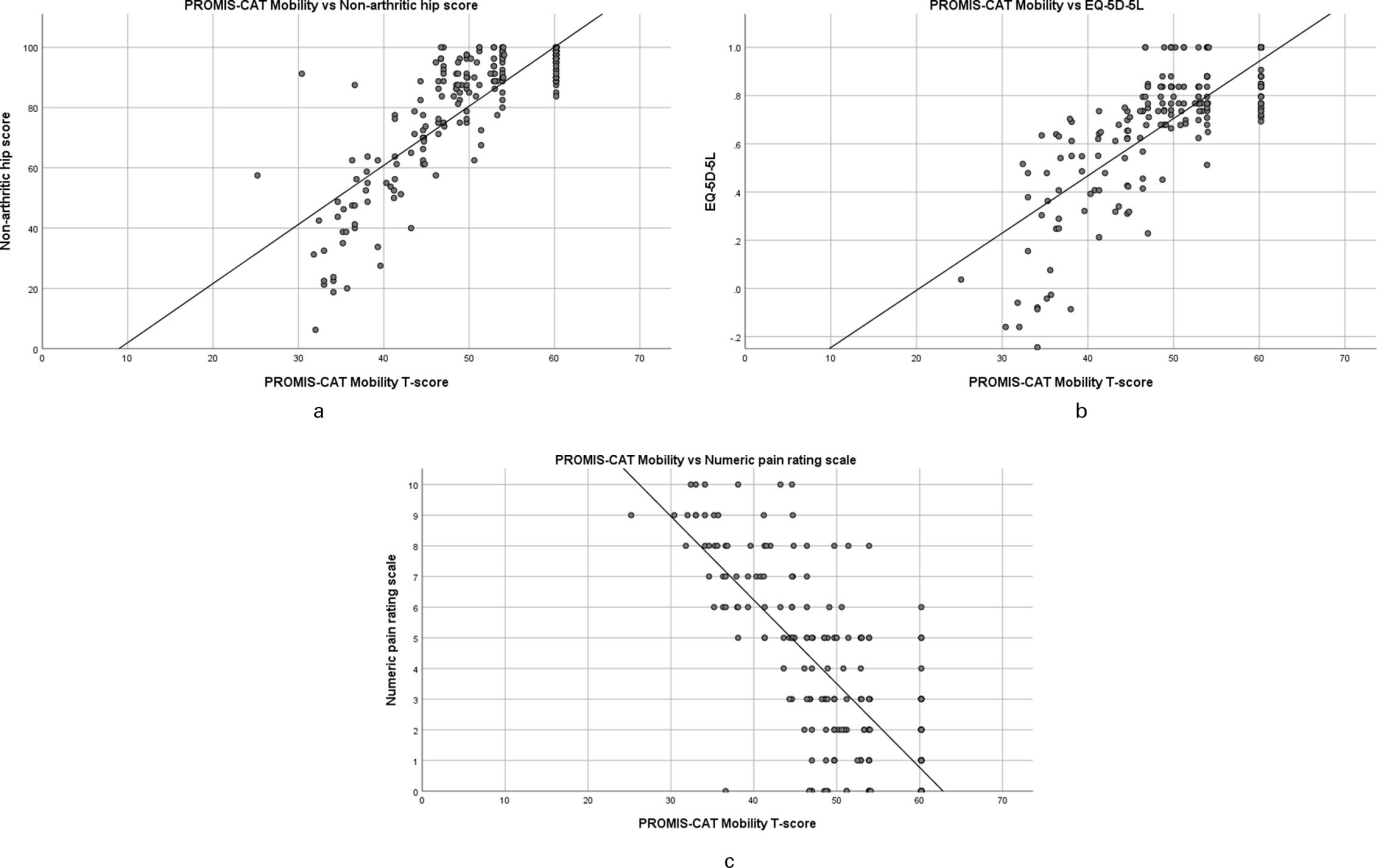
a) Scatter plot of Patient-Reported Outcomes Measurement Information System computer adaptive test (PROMIS-CAT) Mobility score versus Non-Arthritic Hip Score. b) Scatter plot of PROMIS-CAT Mobility versus EuroQol five-dimension five-level. c) Scatter plot of PROMIS-CAT Mobility versus Numeric Pain Rating Scale.

The PROMIS Mobility score showed a strong correlation with the NAHS (*r_s_* = 0.79), as well as other health status measurement tools (EQ-5D-5L; *r_s_* = 0.77, NRS *r_s_* = -0.74) (Table III). Scatter plots of PROMIS Mobility compared to NAHS and other outcome measures were plotted to illustrate this relationship (Figures 1a to 1c). On all plots, an interval with no values was noted in the upper range of PROMIS Mobility T-scores compared to other outcome measures.

**Table III.** Spearman correlation coefficients (r_s_) of the patient-reported outcome measure scores and 95% confidence intervals.

Variable	PROMIS-CAT Mobility (95% CI)	Non-arthritic hip score (95% CI)	EQ-5D-5L (95% CI)
NAHS	0.79 (0.74 to 0.84)		
EQ-5D-5L	0.77 (0.71 to 0.82)	0.86 (0.81 to 0.89)	
NPRS	-0.74 (-0.79 to -0.67)	-0.88 (-0.91 to -0.84)	-0.82 (-0.86 or -0.77)

Correlation is significant at p < 0.001 for all values (two-tailed).

CI, confidence interval; EQ-5D-5L, EuroQol five-dimension five-level; NAHS, Non-Arthritic Hip Score; NRS, Numeric Pain Rating Scale; PROMIS-CAT, Patient-Reported Outcomes Measurement Information System Computer Adaptive Test.

The strength of the correlation between PROMIS Mobility T-score and the subset domains of the NAHS demonstrated that the strongest correlation was physical function and the weakest area was mechanical symptoms: function *r_s_* = 0.81 (95% Cl 0.76 to 0.85; p < 0.001), pain *r_s_* = 0.75 (95% Cl 0.68 to 0.80; p < 0.001), activity *r_s_* = 0.75 (95% Cl 0.68 to 0.80; p < 0.001), and mechanical symptoms *r_s_* = 0.69 (95% Cl 0.61 to 0.75; p < 0.001).

**Fig. 2:**
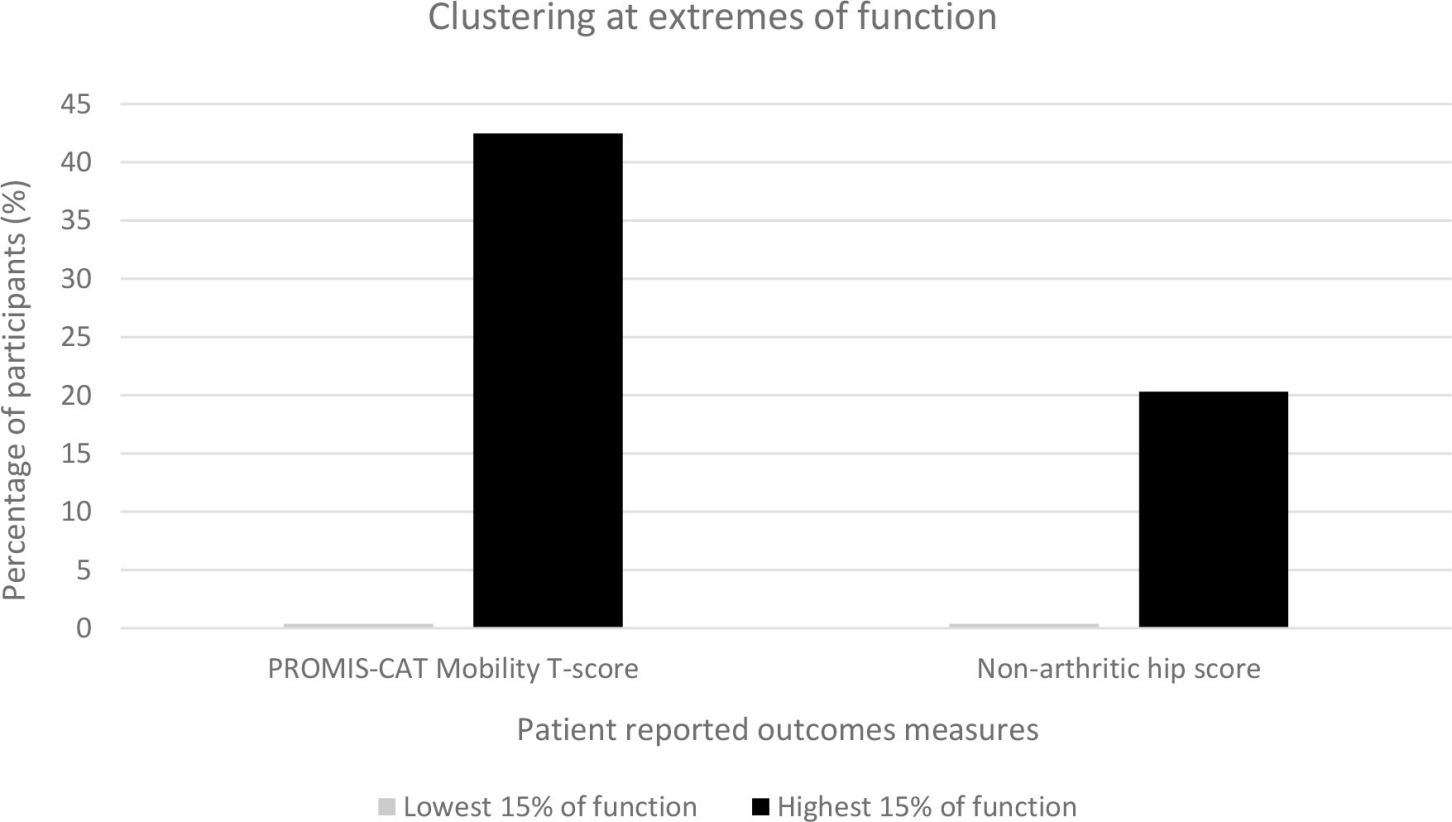
Clustering of the PROMIS-CAT (Patient-Reported Outcomes Measurement Information System computer adaptive test) Mobility score and Non-Arthritic Hip Score.

**Fig. 3:**
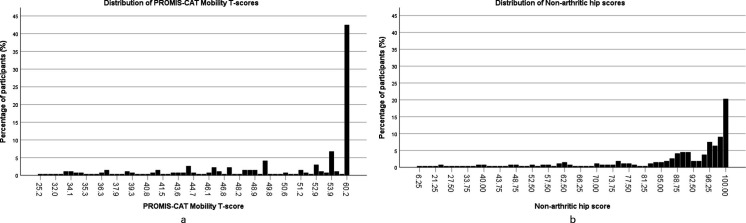
a) Histogram showing the score distribution of Patient-Reported Outcomes Measurement Information System computer adaptive test (PROMIS-CAT) Mobility T-scores. b) Histogram showing the score distribution of Non-Arthritic Hip Score.

Clustering at the extremes of the distributions was assessed for PROMIS and the NAHS (Figure 2). Both showed clustering at the upper extreme of the distributions, but not at the lower extreme. The largest degree of clustering at the upper extreme was seen in PROMIS Mobility score (42.5% of respondents) compared to NAHS (20.3% of respondents). Clustering was also assessed for the domains of the NAHS. All domains also showed notable clustering at the upper extremes of the distribution (function 53.6%, pain 47.7%, mechanical 29.7%, activity 35.0%), and no clustering at the lower end of the distribution. Histograms demonstrated the clustered score distribution for each instrument (Figure 3).

The NAHS minimal clinically important difference (MCID) has been reported to be 8.7.^24^ Assuming that the relationship between the NAHS and PROMIS Mobility is linear; as it broadly appears, the MCID for PROMIS Mobility is approximately 4.2 points. This is consistent with reports in previously published literature.^25^

## Discussion

Scores from PROMIS Mobility were strongly correlated to NAHS in mature patients with a history of childhood hip disease. However, unlike previous studies in hip disease**,** we find notable clustering at the upper extreme of the of PROMIS Mobility score distribution in this population.^26-28^

The NAHS was specifically designed to assess hip function, whereas PROMIS Mobility was designed to assess global lower limb mobility. Therefore, we analyzed subsidiary components of the NAHS to identify those that correlated better with PROMIS Mobility. In particular, we hypothesised that the NAHS subdomains of physical function and activity would have similar constructs to PROMIS Mobility, and would therefore correlate better than the domains of mechanical symptoms and pain. While all domains exhibited relatively strong correlations, as hypothesized, the strongest correlations were in those with areas with similar constructs. The clustering of scores at the upper end of the PROMIS mobility score distribution was broadly comparable to the function and activity domains of the NAHS. PROMIS Mobility did not therefore well capture ‘hip-specific’ characteristics (i.e. pain and mechanical symptoms), which was to be expected given construct and development of the tool.

It is clear from the data distribution that there are no floor effects when using the PROMIS Mobility tool in this population. However, clustering at the upper extreme of the distribution may indicate a ceiling effect. Ceiling effects in other PROMIS tools have been suggested in some adult and paediatric populations. However, we have carefully avoided the term ceiling effect to now. The problem in determining whether clustering around the highest point is due to a ceiling effect is whether the values of the cases actually "represent" the value. When ceiling effects do occur, some of the cases, despite assuming the maximum value, are actually higher than the maximum value (i.e. a child and a surgeon both finish a simple anatomy test to measure one’s ability, both scoring 100%; data is censored thereby there is a ceiling effect). However, in the case of the PROMIS Mobility tool measuring the sequelae of childhood hip disease, it is entirely possible that those who scored the highest are indeed appropriately assigned that score (i.e. for many individuals, outcomes are excellent with no discernible difference in function among these individuals). In this situation, the data is truncated at the limits, not censored; this does therefore not represent a ceiling effect in the outcome tool. Nevertheless, there is an interval at the upper extreme of PROMIS Mobility between which no scores were recorded (Figures 1a to 1d). Items may therefore not exist within PROMIS tools to adequately differentiate between those with the very highest levels of function.

A key advantage of using PROMIS is its efficiency, with an average of 8.5 questions to determine the outcome (though as few as three), compared with a fixed 20 questions within the NAHS. This efficiency is combined with the robustness associated with the development and validation of the tool. The estimate of 4.2 as the MCID for PROMIS Mobility is broadly in keeping with the estimates for PROMIS tools, which is generally 2.0 to 5.0.^25,29^

While comparisons with more legacy instruments could generate a broader comparison, the selected legacy instrument (NAHS) is the most commonly reported in literature for the young, active, and non-arthritic hip patient. To generate a broader clinical picture, we have also included other general health status measurement tools. While there was a low response rate to invitation, the nature of the comparison (correlation of PROMs for an individual, rather than comparing individuals) means that responder bias is unlikely to introduce bias.

In conclusion, we find that the PROMIS Mobility score exhibits strong correlations with the NAHS, and other health quality of life measures in the assessment of lower limb function in our study population. There is a marked clustering of data at the upper extreme, though this seems more likely to represent truncation of data, rather than a ceiling effect. PROMIS Mobility appears to have convergent construct validity in evaluating mobility/physical function. However, there are elements of hip disease not captured within PROMIS Mobility, which is unsurprising given the construct of the tool. This knowledge is helpful in planning clinical trials, where we suggest it may be used to record self-perceived mobility characteristics with additional instruments used to capture other domains within the COS.

**Take home message**

- Patient-Reported Outcomes Measurement Information System (PROMIS) Mobility score demonstrated convergent construct validity for measuring physical function for adolescents and adults with a history of childhood hip disease.

- PROMIS Mobility has truncation of data at the upper extreme of the distribution, which is likely to be a feature of the population, rather than a ceiling effect.
